# The first confirmed case of COVID-19 in Tanzania: recommendations based on lesson learned from China

**DOI:** 10.1186/s41182-020-00214-x

**Published:** 2020-04-26

**Authors:** Clifford Silver Tarimo, Jian Wu

**Affiliations:** 1 College of Public Health, Zhengzhou University, 100 Kexue Avenue, Zhengzhou, 450001 Henan China; 2 Dar es Salaam Institute of Technology, P.O. Box 2958, Dar es Salaam, Tanzania

**Keywords:** Coronavirus disease, Tanzania, China, Recommendations

## Abstract

As the coronavirus disease (COVID-19) takes its course outside China, the number of imported cases to other countries is on the rise. Currently, Tanzania has received the first imported case on 16 March 2020. This letter serves to describe this specific event and put forth a number of recommendations including establishing more testing points, discouraging all forms of public and religious gatherings, and tailoring a unique model for lockdown and social distancing to prevent further spread of the disease. The letter has also suggested ways to further improve the efforts already in place so as to strengthen the prevention and control system.

To the Editor:

Since the coronavirus disease (COVID-19) outbreak was declared a public health emergency of international concern (PHEIC) on 30 January 2020 and yet the pandemic disease, its epidemiology has been reported to change rapidly as pointing to over 118,000 cases in over 110 countries [1–3]. For the first time, since the onset of symptoms of the first identified case of COVID-19 on 8 December 2019, there has been a 13-fold increase in cases reported from countries outside China [4, 5]. As of 16 March 2020, 10:00 AM CET, there were 81,077 confirmed cases and 3218 deaths in China while the authorities outside China’s borders had reported 86,434 confirmed cases, 3388 deaths in 151 countries and thousands more fighting for their lives in hospitals [5]. The statistics tells us that even countries with advanced health systems are still struggling to cope with this epidemic. As the number of cases continues to rise outside China, moving to low-income countries, we should be deeply concerned about the impact it can pose to such population which for decades is characterized by high HIV prevalence [6], chronic non-communicable diseases [7], and malnourished individuals [8]. I think it is high time to report newly recorded incidences and provide clear recommendations while reflecting on China’s experience. On 16 March 2020, the Ministry of Health of Tanzania announced the first case of COVID-19. The victim happened to be a female traveler aged 46 years who departed the country on 3 March 2020 to Belgium and had visited Denmark and Sweden between the dates 5 and 13 March 2020. On the 15 March 2020, the lady flew back to Tanzania from Belgium and arrived at the Kilimanjaro International Airport (KIA) at 1600 hours using the Rwandan airplane. As of other countries, Tanzania had also prepared for the reception of travelers from abroad in terms of diagnostics and trained health care providers and much more. A victim was effectively screened for the symptoms of COVID-19 at the airport and showed none. She took a cab all the way to the hotel located about 28 miles away from the airport. On the 16 March 2020, she felt unwell and decided to surrender herself to medical tests on the same day just to find out that she was infected with the novel severe acute respiratory syndrome coronavirus-2 (SARS-CoV-2). She came to put it clear to the authorities that the reason behind staying at the hotel overnight instead of going home directly was a need for self-quarantine. She further uncovered that, back in Belgium, she was accommodated by her female friend whose husband had acquired the infection. An important thing to learn from this brief story is there is a questionable strategy for screening for this disease as it can obviously miss the truly affected individuals. In addition, body temperature assessment might not be an adequate or sufficient attribute for the screening of COVID-19 [9]. The situation might also mean Tanzania may have a number of imported cases currently large, posing a huge threat to public health. In this regard, I thought it is imperative to bring forth some few recommendations:

(i) As the Ministry of Health of Tanzania suggests, there is no need to panic. In this period when most countries worldwide are taking measures to prevent the importation of the virus, the government of Tanzania should improve in monitoring the disease epidemiology by improving strategies for identification, diagnosis, management of cases, contact tracing, and implementation of strict measures for returning travelers.
(ii) As travelers coming from risky countries are quarantined for the recommended time (not less than 14 days) while undergoing symptoms based as well as laboratory tests, the government should make a significant improvement in establishing more resources and equipment for quarantine and isolation. If this approach is effectively applied, it can dramatically slow down the spread as it was appreciated in China where strict restrictions were put in place to quarantine nearly 60 million people in Hubei province that led to a tremendous decline of new cases from nearly 1600 cases each day to less than 40 cases [10].
(iii) The government should build capacity to the existing healthcare centers to diagnose the infection instead of depending on the national laboratory located in Dar es Salaam.
  In addition, as the cases are still accumulating, one of the cost-effective approaches may be to transform some of the existing structures such as malls or exhibition centers and prepare them to receive COVID-19 patients. Establishment of safe rooms for negative-pressure rooms (contagious respiratory disease isolation) to accommodate patients who might require more sophisticated medical attention should also be considered.
(iv) In case of symptoms suggestive of acute respiratory illness before, during, or after travel, the travelers are encouraged to seek medical attention and share travel history with their health care providers. In addition, public health authorities of Tanzania should provide to travelers information to reduce the general risk of acute respiratory infections, via health practitioners, travel health clinics, travel agencies, conveyance operators, and at points of entry.
(v) As panic still prevails in Tanzania and the rest of African countries, we should all beware of infodemic which may hamper the efforts to fight against the virus. Currently, there have been a lot of misinformation and contradicting facts that are posted especially through the social media networks in Tanzania. The citizens of Tanzania should be encouraged to rely only on the trusted and reliable sources such as the Ministry of Health, Center of Disease Control and prevention (CDC), and the World Health Organization (WHO) as it is in China.
(vi) This letter calls upon the government of Tanzania to immediately suspend not only schools and colleges as it has been successfully done but also all religious and any other social gatherings. In China, all unnecessary or non-essential large-scale public gatherings were stopped as early as 25 January 2020 [11]. The application of this approach can provide significant contribution towards flattening the epidemic curve.
(vii) As it has been shown by WHO and well-practiced in China during the epidemic, the use of banknotes should be banned [12]. I advise the ministry of finance of Tanzania to encourage citizens to use contactless payment such as mobile applications and electronic bank transactions by ensuring ease of its use, security, and privacy. Despite the announcement from the Bank of Tanzania that the banknotes cannot harbor the virus [13], CDC has acknowledged that banknotes in circulation can hold virus and bacteria and hence recommends rigorously that people wash their hands after handling money [12]. In addition, a study conducted in 2017 revealed a list of things including DNA from pets, traces of drugs, bacteria, and viruses found on banknotes [14]
(viii) In response to COVID-19 epidemic, the central government of China imposed a lockdown in the epicenter, Wuhan city of Hubei province. However, this lockdown model might not work in low- and middle-income settings like Tanzania. This has been proven in other East African countries like Kenya where there have been episodes of civil unrest as citizens are enforced to stay home. In this regard, the government of Tanzania is called to come up with the African-based lockdown and social distancing model that will specifically suit the citizen. This is mainly because a significant proportion of Tanzanians have to work daily in the streets to earn a living.
(ix) Since this crisis is proving to be more difficult and longer-lasting than any of us had expected, there is a need for Tanzania to formulate a rapid network with Chinese doctors and health care providers that will ensure a rapid sharing of information and how to effectively and timely diagnose the viral infection and track cases. By joint cooperation, we are more than capable of containing the virus.

## Data Availability

Data used to draw the conclusion are provided in this letter. Conclusions drawn in this letter reflect the authors’ views and not the Institution of Affiliation.

## Abbreviations

CET: Central European Time
COVID-19: Coronavirus disease-2019
CDC: Center for Disease Control and Prevention
HIV: Human immunodeficiency syndrome
KIA: Kilimanjaro International Airport
PHEIC: Public health emergency of international concern
SARS-CoV-2: Severe acute respiratory syndrome coronavirus 2
WHO: World Health Organization
